# Proteostasis (in)action: the role of co-pathologies in neurodegenerative disease

**DOI:** 10.1098/rstb.2025.0273

**Published:** 2026-10-01

**Authors:** James F. Morley

**Affiliations:** ^1^ Parkinson's Disease Research, Education and Clinical Center, US Department of Veterans Affairs, Philadelphia, PA, USA; ^2^ Neurology, University of Pennsylvania, Perelman School of Medicine, Philadelphia, PA, USA

**Keywords:** neurodegeneration, proteostasis, co-pathology

## Abstract

Clinically distinct neurodegenerative diseases, including Alzheimer's disease (AD), Parkinson's disease (PD) and amyotrophic lateral sclerosis (ALS), share in common progressive neuronal dysfunction and cell death associated with the accumulation of misfolded proteins. Central to these processes are the cellular and organismal proteostasis networks responsible for maintaining protein homeostasis through coordinated actions of molecular chaperones, the ubiquitin–proteasome system and autophagy–lysosomal pathways. As a consequence of ageing, genetic modifications and other disease-specific conditions, the proteostatic network becomes compromised, leading to the accumulation of toxic protein aggregates that disrupt neuronal function and lead to neurodegeneration. Specific misfolded proteins are associated with each neurodegenerative disease (i.e. α-synuclein in PD, amyloid-β/tau in AD and TAR DNA-binding protein 43/SOD in ALS). However, increasing evidence implicates more complex interactions of co-pathologies across these disorders, as Lewy bodies are common in AD brains and Alzheimer pathology is present in the majority of PD autopsy cases. These observations are consistent with the idea that disruption of proteostasis networks by one aggregation-prone protein could result in misfolding and aggregation of other neurodegeneration-related species. This review will examine the characteristics and consequences of co-pathologies in neurodegenerative disorders. Additionally, the review will outline emerging therapeutic strategies targeted at restoring proteostasis and mitigating the effects of co-pathological processes associated with neurodegenerative diseases.

This article is part of the Theo Murphy meeting issue ‘ProteostaSys: a systems view of proteostasis’.

## 1. Phenotypically diverse neurodegenerative disorders are linked by the appearance of misfolded and aggregated proteins

Neurodegenerative diseases represent a clinically diverse group of disorders unified by a common pathological feature: the misfolding and aggregation of specific proteins into toxic conformations that result in characteristic inclusions in the brain [1–3]. Alzheimer's disease (AD), Parkinson's disease (PD), amyotrophic lateral sclerosis (ALS) and frontotemporal lobar degeneration (FTD) exhibit distinct clinical presentations but share this fundamental pathologic mechanism. Additionally, each of these disorders is characterized and pathologically defined by the aggregation of characteristic proteins—amyloid-β and tau in AD, α-synuclein in PD and TAR DNA-binding protein 43 (TDP-43) in ALS [4–6]. These proteins undergo conformational changes from their native states to form oligomers and ultimately insoluble fibrils that deposit as plaques, tangles, Lewy bodies and neurites, or other inclusions. Deposition of these inclusion bodies in disease-specific brain regions results in the characteristic clinical presentations. Despite involving distinct proteotoxic species, misfolding and aggregation follow remarkably parallel pathways across these disorders, with increasing evidence suggesting that oligomeric intermediates, rather than end-stage fibrils or inclusions, are the toxic species mediating neuronal dysfunction and death [7,8].

Neurodegenerative diseases associated with protein misfolding and aggregation ultimately reflect a failure of proteostasis—the cellular machinery responsible for maintaining proteins in their correct conformation, concentration and localization [9]. A network of quality-control mechanisms, including molecular chaperones, the ubiquitin–proteasome system and autophagy-lysosomal pathways, monitors protein misfolding and eliminates aberrant proteins before they can aggregate, maintaining proteostasis under normal conditions. However, perturbation of proteostasis through genetic mutations, post-translational modifications, oxidative stress and the age-related decline in proteostatic capacity can overwhelm these protective systems [8,10]. When chaperone function is overwhelmed, or other clearance mechanisms fail, misfolded proteins accumulate and aggregate, triggering a cascade of cellular responses that ultimately lead to cell dysfunction and progressive neuronal loss [11].

## 2. Co-occurrence of pathologies in neurodegenerative disease is common, heterogeneous and related to disease characteristics

Although each clinical syndrome is historically associated with a particular cognate protein, it is increasingly clear that neurodegenerative pathologies regularly occur together rather than in isolation. In one study of 766 autopsied individuals with primary neurodegenerative dementia pathologies, including tau, amyloid-β, α-synuclein and TDP-43, having multiple pathologies was more common than ‘pure proteinopathy’ alone [12]. Tau co-pathology was most common, appearing in more than 90% of cases [12]. The frequency of co-pathology in most cases was similar in the dementia cohort to that observed in a group of age-matched controls. However, increased frequency of co-pathology was observed in neocortical Lewy body disease (amyloid-β and TDP-43), and AD (α-synuclein and TDP-43), suggesting that co-pathology did not simply represent co-occurrence by chance [13]. Cognitive and motor decline was faster in Lewy body subjects with co-pathology, suggesting an effect on disease progression [12].

In a follow-up study, Robinson *et al.* [14] studied 10 different brain pathologies across eight different clinical groupings (including ‘normal ageing’) in 1647 autopsied individuals. The authors identified up to seven co-morbid pathologies in any individual and a total of 161 different observed pathologic combinations among all individuals. Consistent with their prior study [12], the authors found that most co-pathologies occurred at rates similar to that seen in controls. However, co-pathology was observed at higher frequencies in Alzheimer and Lewy body disease compared with ‘normal ageing’ [14]. In an accompanying commentary, Younes & Mormino [15] observe that, within a particular clinical diagnosis, patients are likely to have individual ‘pathotomes’ that will be necessary to understand disease heterogeneity and possibly offer opportunities for using precision medicine-based therapeutic strategies.

## 3. Co-incident Alzheimer and Lewy pathology: characteristics and consequences

Perhaps the best studied pairing of co-pathologies in neurodegenerative disease is the co-occurrence of Lewy and Alzheimer pathology. α-synuclein–based Lewy pathology is common in AD brains, appearing in the majority (approx. 50%–60%) of autopsy-proven cases, with a predominance in limbic areas [16–18]. Additionally, there is regional specificity in the association between α-synuclein deposition and higher amyloid-β burden, particularly in the amygdala [19]. AD cases with higher cortical α-synuclein deposition have younger age at death, and the amygdala-predominant α-synuclein pattern associates with increased cortical tau load, whereas brainstem-predominant and cortical α-synuclein cases show decreased cortical tau compared with α-synuclein-negative AD. Additionally, higher α-synuclein burden is associated with male sex, younger age at onset and more severe neuropsychiatric burden in AD [20–22]. Importantly, AD-associated genetic variants, including apolipoproteinE (APOE) ε4 and presenilin, are associated with higher Lewy pathology as well as cortical amyloid-β deposition in AD, suggesting shared genetic risk factors for multiple proteinopathies [19,23–26].

Similarly, Alzheimer's pathology is remarkably common in autopsy-proven Lewy body disorders. In fact, approximately 65% of all Lewy body dementia cases *also* meet AD neuropathological criteria [27–29]. The prevalence of AD pathology in Lewy body disorders is associated with phenotypic differences, as nearly 80% of dementia with Lewy bodies (DLB) cases harbour intermediate-to-severe AD co-pathology, whereas only 50% of PD dementia (PDD) cases show moderate-to-severe cortical and hippocampal amyloid deposition [29]. There are hints at biochemical differences as well because the tau pathology in DLB appears less compact than that observed in PDD [29]. Lewy body disorder patients with co-morbid AD pathology exhibit accelerated cognitive decline, shorter intervals between motor symptom onset and dementia, and reduced survival [12,27,30,31]. Reminiscent of the regional specificity of synuclein deposition in AD, the distribution of tau differs among clinical phenotypes, with Lewy body disease (LBD)-AD showing greater proportional tau burden in temporal neocortex and AD showing frontal predominance [29].

## 4. Is co-pathology more than chance?

Some co-pathologies occur at similar frequencies to those observed in healthy controls. However, others, such as co-occurrence of AD and Lewy pathology, occur at higher frequencies than in the general population, suggesting that the emergence of one pathology facilitates the appearance of another. One reasonable hypothesis, as discussed in detail by other papers in this issue, is that the challenge, and ultimate failure, of proteostatic networks caused by one aggregation-prone protein ‘opens the floodgates’ for others. There are multiple mechanisms by which proteostasis failure could occur under these circumstances. For example, it has been proposed that one misfolding-prone protein could challenge proteostasis by: (i) misfolding of one protein lowering the threshold for global misfolding; (ii) inhibition of proteostasis components causing global aggregation phenomena; and (iii) proteome-wide risk of protein aggregation (which has been described in the ‘supersaturation’ or ‘phase transition’ literature around protein aggregation).

However, emerging evidence suggests that α-synuclein, tau and amyloid-β can also *directly* promote one another's aggregation through multiple mechanisms. In one study, injection of α-synuclein preformed fibrils into mice with already abundant amyloid-β plaques accelerated local synuclein pathology and spread throughout the brain, also inducing hyperphosphorylated tau [32]. Structural studies have demonstrated that Aβ42 aggregates into fibrillar assemblies that can serve as nucleation surfaces facilitating initiation of α-synuclein aggregation [33,34]. Multiply-transgenic mice expressing mutant α-synuclein crossed with mice harbouring Aβ plaques and/or tau tangles demonstrate enhancement of all three pathologies and accelerated cognitive decline compared with mice with only one of the proteinopathies [35,36]. Either through failure of proteostatic networks or direct effects, such interdependent interactions facilitate pathological cascades that accelerate neurodegeneration beyond what occurs with single proteinopathies. Although *in vitro* studies strongly suggest that direct inter-molecular interactions may facilitate cross-seeding leading to co-pathology [33,34], mechanistic kinetic studies of protein aggregation in human brains are inherently limited, and indirect effects of amyloid on synuclein pathology (and vice versa) cannot be ignored.

## 5. Obstacles and opportunities for therapeutics targeting proteostasis

Strategies targeting proteostasic mechanisms are in various stages of development, including enhancing autophagy, modulating molecular chaperones and blocking protein aggregation. Autophagy-based approaches are the most studied among these to date [37]. Small molecules targeting mTOR, including rapamycin and sirolimus, have been shown to reduce pathology in preclinical models and are being tested in clinical trials for AD, Huntington's disease, ALS and PD [38,39]. Other compounds that target autophagy, such as trehalose, spermidine and lithium, are also under investigation, with spermidine being evaluated for memory performance in older adults [40,41].

Chaperone-based therapies can facilitate direct refolding of misfolded and aggregation-prone proteins, deliver them to degradation pathways, or reduce aggregates to species that can then be cleared via autophagy [42]. Arimoclomol, a co-inducer of heat shock protein expression, reduced neuronal death, improved neuromuscular function and extended lifespan in a SOD1 mouse model of ALS [43]. Additionally, arimoclomol was shown to reduce ALS/FTD-like phenotypes and prevent cell death in an ALS mouse model and can ameliorate pathology in patient fibroblasts and induced pluripotent stem cells-derived motor neurons [44]. Direct aggregation inhibitors and proteasome activators represent additional strategies [45].

Most recently, antibody-based strategies directly targeting aggregation-prone proteins have emerged as promising possibilities. Monoclonal antibodies against amyloid-β and α-synuclein have been successfully developed in animal models and recently tested in human clinical trials. Two antibodies (Lecanemab and Donanemab) have been recently approved by the United States Food and Drugs Administration for treatment of AD on the basis of their ability to reduce amyloid positron emission tomography accumulation and provide modest clinical improvement [46–49]. A phase 2 clinical trial of prasinezumab, a monoclonal antibody targeting aggregated α-synuclein, failed to meet its primary endpoints but subgroup analyses suggest it may slow motor progression in a subset of individuals and will be studied in an upcoming phase 3 trial [50–52]. Work on these and an expanding list of antibody therapies continues but the currently available tools suggest strategies to potentially exploit co-pathology to prevent certain disease complications. For example, cognitive impairment leading to dementia is perhaps the most feared feature of PD, but typically does not occur until relatively late in the course and is associated with the appearance of Alzheimer pathology [53]. Trials are currently being designed whereby anti-amyloid antibodies will be tested in early, cognitively normal PD to test whether progressive cognitive impairment can be delayed or prevented.

Unfortunately, these promising therapies, with the possible exception of antibody therapies, have had limited success in human trials. There are many challenges that limit clinical translation of these approaches. For example, autophagy-based approaches require functional lysosomes, which may be impaired in diseases like AD and ALS; in such cases, enhancing autophagy without addressing lysosomal dysfunction could worsen disease [54]. Additionally, many autophagy or chaperone-modulating drugs lack specificity and affect multiple cellular pathways. Promising treatments from preclinical models (often given throughout the life of the animal) may be ineffective in the setting of clinically manifest disease with an already high burden of pathology, motivating strategies to improve early or even prodromal diagnosis using clinical, fluid and radiologic biomarkers. Although patients with a given disease may appear clinically similar, the underlying molecular mechanisms are often diverse (e.g. PD caused by *GBA*, *parkin* or *Pink1* mutations that all affect different pathways) such that a clinical trial with a drug targeting a single target may not benefit many subjects whose disease is driven by alternative pathways, again highlighting the importance of biomarker development and stratification for clinical trials [55].

## 6. Summary

Neurodegenerative diseases such as AD, PD, ALS and FTD are linked by the misfolding and aggregation of specific proteins, including amyloid-β, tau, α-synuclein and TDP-43. The appearance of toxic misfolded species reflects failure of proteostasis, the cellular mechanisms that normally maintain protein homeostasis. Co-occurrence of multiple pathologies within individual cases is common and influences clinical characteristics and disease progression. Co-morbid Alzheimer and Lewy pathology, for example, exacerbates disease progression, resulting in more severe symptoms and faster cognitive decline in both PD and AD. Understanding the interdependent mechanisms of protein aggregation to develop targeted therapies to modulate proteostatic pathways for treatment offers important opportunities but, to date, has faced challenges in clinical translation. Future studies using biomarker stratification and targeting earlier interventions are likely to improve the success of targeting proteostatic networks in neurodegenerative disease (table 1).

**Table 1.** Summary of clinicopathological correlations in human autopsy-based studies of co-pathology in neurodegenerative disease.

	**diagnoses/pathologies studied**	**sample size**	**pathological findings**	**clinicopathological correlations**
Lippa *et al.* [23]	AD, DLB, MTL atrophy	80	medial temporal lobe (MTL) atrophy lower in DLB vs AD	DLB retains memory better than AD, less hippocampal involvement
Lippa *et al.* [24]	AD, DLB, Aβ40/42 levels	46	different β-amyloid ratios distinguish AD from DLB	amyloid profiles linked to neuropsychological features (amnestic)
Hamilton [16]	Lewy bodies (a-syn) in AD	145	63% of AD had Lewy bodies; variable distribution, most in amygdala	more parkinsonism/fluctuating cognition with both pathologies
Duda *et al.* [28]	a-syn, tau in genetic PD	5 families	all confirmed cases had both alpha-synuclein and tau	co-pathology may modify clinical phenotype in genetic PD/DLB
Leverenz *et al.* [25]	familial AD, LBD	39	pathology varies by mutation; LBD frequent in PSEN1	varying clinical courses by co-pathology and gene mutation
Schneider *et al.* [17]	AD, LBD, vascular, TDP-43	483	high rates mixed pathologies (>50%); LBD frequent in AD; vascular common in all	mixed pathologies linked to more severe cognitive impairment
Jicha *et al.* [21]	Lewy, AD pathology in clinical LBD	21	pure and mixed LB pathology observed	prodromal symptoms better predicted by presence of co-pathology
Tsuang *et al.* [26]	AD, Lewy pathology in AD, PD, PDD, DLB	640	APOE4 increases dementia risk in synucleinopathies with AD	greater cognitive decline in synucleinopathies with AD co-pathology
Chung *et al.* [22]	AD with and without Lewy bodies	531	41% of AD cases had Lewy bodies	AD+LBD had more parkinsonism/hallucinations vs pure AD
Toledo *et al.* [18]	AD and Lewy pathology in AD, PD, DLB	215	wide variation in alpha-synuclein distribution in AD+LBD	presence of both associated with more severe overall neuropathology and disease progression
Irwin *et al.* [31]	AD, tau in synucleinopathies,	213	AD and tau co-pathology common in synucleinopathies	faster dementia onset, reduced survival with higher AD/tau pathology
Robinson *et al.* [12]	AD, LBD, PSP, CBD, TDP-43	766	multiple co-pathologies more common than single pathologies	greater co-pathology associated with lower cognition and older age
Savica *et al.* [20]	Lewy pathology in AD	187	43% of AD had Lewy bodies	AD+LBD had worse motor function and more rapid cognitive decline than pure AD
Coughlin *et al.* [27]	AD pathology in LBD	80	tau pathology in LBD varies widely in distribution and severity	higher tau load in LBD associated with worse cognitive impairment
Robinson *et al.* [14]	AD, LBD, TDP-43, PSP and normal ageing	1493	pathological combinations are diverse and disease-specific (161 different combinations)	distinct clinicopathological profiles by co-pathology, higher overall burden of co-pathology than expected
Neubauer *et al.* [19]	a-syn, Aβ, tau in AD	91	a-syn correlated with amyloid and tau load	higher co-pathology associated with worse cognitive decline, reduced survival

Abbreviations: AD, Alzheimer's disease; LBD, Lewy body disease; DLB, dementia with Lewy bodies; PSP, progressive supranuclear palsy; CBD, corticobasal degeneration; TDP-43, TAR DNA-binding protein 43; PDD, Parkinson's disease dementia; MSA, multiple system atrophy; a-syn, alpha-synuclein.

## Data Availability

This article has no additional data.

## Theme

One contribution of 20 to a Theo Murphy meeting issue ‘ProteostaSys: a systems view of proteostasis’.
